# Cutpoints for mild, moderate and severe pain in patients with osteoarthritis of the hip or knee ready for joint replacement surgery

**DOI:** 10.1186/1471-2474-9-55

**Published:** 2008-04-21

**Authors:** Heidi Kapstad, Berit R Hanestad, Norvald Langeland, Tone Rustøen, Knut Stavem

**Affiliations:** 1 Oslo University College, Faculty of Nursing Education, Oslo, Norway; 2 University of Bergen, Department of Public Health and Primary Health Care, Bergen, Norway; 3 Department of Orthopaedics, Rikshospitalet-Radiumhospitalet Medical Centre, Oslo, Norway; 4 The Norwegian System of Compensation for Patients (NPE), Oslo, Norway; 5 Centre for Shared Decision Making and Nursing Research, Rikshospitalet-Radiumhospitalet Medical Centre, Oslo, Norway; 6 Medical Department, Akershus University Hospital, Lørenskog, Norway; 7 Helse-Øst Health Services Research Centre, Lørenskog, Norway; 8 Faculty of Medicine, University of Oslo, Norway

## Abstract

**Background:**

Cutpoints (CPs) for mild, moderate and severe pain are established and used primarily in cancer pain. In this study, we wanted to determine the optimal CPs for mild, moderate, and severe pain in joint replacement surgery candidates with osteoarthritis (OA) of the hip or knee, and to validate the different CPs.

**Methods:**

Patients (n = 353) completed the Brief Pain Inventory (BPI), the WOMAC Arthritis Index, and the SF-36 health status measure. Optimal CPs for categorizing average pain with three severity levels were derived using multivariate analysis of variance, using different CP sets for average pain as the independent variable and seven interference items from the BPI as the dependent variable. To validate the CPs, we assessed if patients in the three pain severity groups differed in pain as assessed with WOMAC and SF-36, and if BPI average pain with the optimal CPs resulted in higher correlation with pain dimensions of the WOMAC and SF-36 than other CPs.

**Results:**

The optimal CPs on the 0–10 point BPI scale were CP (4,6) among hip patients and CP (4,7) among knee patients. The resulting pain severity groups differed in pain, as assessed with other scales than those used to derive the CPs. The optimal CPs had the highest association of average pain with WOMAC pain scores.

**Conclusion:**

CPs for pain severity differed somewhat for patients with OA of the hip and knee. The association of BPI average pain scores categorized according to the optimal CPs with WOMAC pain scores supports the validity of the derived optimal CPs.

## Background

Patients with osteoarthritis (OA) of the hip or knee can experience pain for several years prior to joint replacement surgery [1-3], and increased pain and decreased mobility are important determinants of when to operate [3,4]. Recently, patients with both cancer [5,6] and non-cancer pain [5,7-9] have been categorised into mild, moderate, and severe pain groups based on pain intensity ratings.

The method for establishing cutpoints (CPs) to categorise pain severity is established for classification of cancer pain into mild, moderate, and severe pain [6]. Using worst pain intensity scores from Brief Pain Inventory (BPI) from 0 (no pain) to 10 (worst pain), ratings from 1 to 4 corresponded to mild pain, 5 to 6 to moderate pain, and 7 to 10 to severe pain [6]. This cutpoint pattern is symbolically represented by (4,6), indicating that the top score for mild pain is 4 and the top score for moderate pain is 6. This severity classification is used to establish treatment algorithms for cancer pain management [10,11]. Subsequent work in non-cancer pain has reported different CPs for phantom limb pain, back pain, and for pain in general [7], and that the optimal CPs for low back pain and a pooled sample of patients with OA are (5,8), and (5,7), respectively [8]. Assessment of the multidimensional aspects of pain is important, and pain severity is the primary factor to determine the impact of pain on the patient for establishing pain treatment, priority in the treatment process, and in the communication between health care providers and patients [12].

In patients with OA, because pain and decreased function are important clinical indicators of the need for surgery, it would be useful to know if specific pain severity categories were associated with poorer outcomes. No study has established CPs for pain severity in homogeneous samples of patients with pain from OA of the hip or knee who are scheduled for joint replacement surgery. Hence, it is unclear if CPs for pain severity are different in patients with OA of the hip or knee.

The aims of the study were (1) to determine the optimal CPs for mild, moderate, and severe pain in patients with OA of the hip or knee, based on patients' ratings of average pain, and (2) conduct an initial validation of the BPI with different CPs, by investigating if the optimal CPs resulted in a higher association with the pain scales of a disease-specific measure of functional disability and a generic health-related quality of life measure, than other cutpoints.

## Methods

### Sample, settings and procedures

This study is part of a large, prospective, longitudinal, multi-center study that evaluated pain and HRQOL outcomes in patients with OA before and after joint replacement surgery. A total of 503 patients who entered the waiting list for hip or knee joint replacement surgery from six different hospitals in the eastern part of Norway were invited to participate in the study. Recruitment took place from June 2003 to June 2004. The participants were adults (> 18 years), who were enrolled on the waiting list for primary joint replacement surgery and able to read, write, and understand Norwegian. In total 353 patients accepted to participate in the study.

In the six hospitals, a nurse or administrative staff member identified potential participants who were placed on the waiting list for a hip or knee replacement and determined whether they met the study's inclusion criteria. All patients who met the inclusion criteria were sent a letter that explained the study, a packet of questionnaires, and a stamped, self-addressed envelope. Participants returned the completed questionnaires, which constituted written informed consent, to the research office.

Among the 353 participants, 318 provided complete data for this analysis. No differences in gender and type of surgery (hip or knee joint replacement) were found between those who did and did not participate in the study. However, participants were younger (69.5 years (SD = 9.3)) than non-participants (72.9 years (SD = 9.0); p < .001). Because this survey was mailed to potential participants, the reasons for not returning the questionnaires are not known. This study was approved by The National Committee for Research Ethics in Norway, the Norwegian Social Science Data Services, and each of the study sites.

### Questionnaires

At baseline, patients completed a questionnaire that obtained data on gender, age, marital status, cohabitation, education level, employment status, type of surgery, duration of pain in the joint, number of years with ambulation problems, and a rating scale on urgency for surgery [13]. In addition, patients completed the Brief Pain Inventory (BPI) [14], the Western Ontario and McMaster Universities Osteoarthritis Index (WOMAC) [15,16], and the Medical Outcomes Study – Short Form (SF-36) [17-19] questionnaires.

### The Brief Pain Inventory

The BPI is a short, self-administered questionnaire designed to evaluate the intensity of pain and the impairment caused by pain during the past 24 hours. Four items measure pain intensity (pain now, average pain, worst pain, and least pain) using 0 ("no pain") to 10 ("pain as bad as you can imagine") numeric rating scales. Seven items measure the level of interference with function caused by pain (general activity, mood, walking ability, normal work, relations with other persons, sleep, and enjoyment of life) using 0 (no interference) to 10 (complete interference) rating scales. In addition, patients rate the amount of pain relief they are experiencing using a 0% ("no pain relief") to 100% ("complete pain relief") rating scale. The Norwegian translation of the BPI, which has satisfactory validity and reliability [20], was used in this study. Originally, the BPI was developed to evaluate cancer pain, but it has been shown to be a valid and reliable instrument for chronic non-cancer pain [21-23].

### The WOMAC Osteoarthritis Index

The WOMAC is a three-dimensional, disease-specific, and self-administered instrument [15,16] that consists of 24 items that evaluate pain (five items), stiffness (two items), and overall level of physical function (17 items). Items are rated using one of five responses (0 = none, 1 = mild, 2 = moderate, 3 = severe, 4 = extreme). Three subscale scores are calculated, pain (0 to 20), stiffness (0 to 8), and physical function (0 to 68).

The WOMAC is a valid, reliable, and sufficiently sensitive instrument that can detect clinically important changes following a variety of interventions for OA [15]. For this study, patients were asked to respond to each item in relationship to the hip or knee joint that was to be replaced. We used the Norwegian Likert scale version 3.1, which assesses pain, stiffness and physical function during the past 48 hours. The questionnaire was translated to Norwegian by a standardized procedure with forward and backward translation and has been used in previous studies [24,25]. The psychometric properties of the WOMAC have been documented in many languages, but this has not yet been reported for the Norwegian version. In this paper, we concentrated on the pain dimension.

### Medical Outcomes Study-Short Form (SF-36)

The SF-36 consists of 36 items that evaluate eight conceptual domains of HRQOL: general health (GH), physical functioning (PF), mental health (MH), role limitations – physical (RP), role limitations- emotional (RE), vitality (VT), bodily pain (BP), and social functioning (SF) during the past 4 weeks [18]. This reliable and valid instrument has been used in numerous studies of HRQOL and in patients with pain from OA [1-3,8,26-31]. The Norwegian translation of the SF-36 was used [32] and scored so that each domain's score ranged from 0 to 100, with higher scores indicating a better HRQOL. The SF-36 is an internationally accepted measure of HRQOL with documented validity and reliability [18,19]. In this paper, we concentrated on the bodily pain dimension.

### Data analysis

Descriptive statistics and frequency distributions were generated for the patients' demographic and disease-related characteristics. Data are presented as means and standard deviations (SD) or frequencies and percentages. Demographic characteristics were compared using the Kruskal-Wallis test or chi square test.

CPs that divided the sample into mild, moderate, or severe pain groups were created for ratings of average pain using the analytic strategy described by Serlin and Mendoza et al. [6]. In this analysis, pain severity categorized according to the cutpoints was the independent variable, and the seven interference items from the BPI were the dependent variables. Hence, we created eight MANOVA models for average pain. Eight different categorical variables, which represented the eight possible cut-off values for the CPs, between 3 and 7, were created and related to the set of seven interference items from the BPI using multivariate analysis of variance (MANOVA). Wilk's lambda is one of the most widely used criteria for statistical testing of MANOVA models, and both the Pillai's trace and Hotelling's trace are alternative criteria that are functions of the eigenvalues.

For example, CPs (4,7), were coded so that a pain severity rating of 1 to 4 would correspond to "mild", > 4 to 7 to "moderate", and > 7 to 10 to "severe" pain. The criterion used to determine the optimal set of CPs for mild, moderate, and severe pain was that a MANOVA among pain severity categories yielded the largest F ratio for the between category effect on the seven interference items as indicated by Pillai's trace, Wilks' lambda, and Hotelling's trace F statistics. To determine the optimal CPs, we used the lowest median rank of the ranking of these three statistics.

We used only the average pain CPs in our analysis, because a rating of average pain may be more representative of the chronic or persistent stable pain associated with a sample of patients with OA [7,8].

To determine if the BPI using different CPs for grading of pain severity, were associated with corresponding scales of the other questionnaires, namely the pain dimension of the WOMAC and the bodily pain scale of the SF-36, we used Spearman's rank correlation. We hypothesized that the optimal CPs would show higher association with these scales than non-optimal cutpoints. A confirmation of this would support the validity of the optimal CPs. Moreover, we would expect the association of the BPI scores with the WOMAC scale to be higher than with the Bodily Pain scale of the SF-36, because of the more similar time perspective in the framing of the questionnaire items.

We assessed if the resulting pain severity groups differed in pain history and pain as assessed with other items of the BPI, which were not used for deriving the CPs. For this analysis, we used one-way analysis of variance (ANOVA) with Bonferroni correction for multiple comparisons.

A p-value of < .05 was considered statistically significant, using two-sided tests. For the ANOVA analyses, the p-value presented for each pairwise contrast was adjusted so that a value of < .05 indicates statistical significance. Data were analyzed using SPSS for Windows version 12.0.

## Results

### Sample characteristics

The majority of the sample was female for both hip (71%) and knee (78%), with a mean age of 68.7 years (SD = 9.7; range 41 to 91 years) for hip and 69.1 years (SD = 8.3; range 47 to 86 years) for knee. In the hip sample, 67% were married/partnered, compared with 59 % in the knee sample. Among the patients with OA of the hip or knee, 67% and 80% had completed at least a secondary school education, respectively. Details about demographic characteristics are shown in Table 1.

**Table 1:** Demographic characteristics among patients with OA of the hip and knee, number (%) unless otherwise stated

	**Patients with OA of the hip** **(n = 224)**	**Patients with OA of the knee** **(n = 94)**
**Age **(years), mean (SD)	68.7 (9.7)	69.2 (8.3)
**Gender **(Female)	160 (71)	73 (78)
**Marital status**		
Single	9 (4)	5 (5)
Married/partnered	149 (67)	55 (59)
Divorced/Separeted	29 (13)	12 (13)
Widowed	37 (16)	22 (23)
**Cohabitation **(Yes)	157 (70)	59 (63)
**Education level**		
Primary school	59 (26)	33 (35)
Secondary school	92 (41)	41 (45)
University < 4 years	40 (18)	10 (11)
University ≥ 4 years	33 (15)	9 (9)
**Employment status**		
Retired	136 (61)	56 (60)
Disability	28 (13)	23 (25)
Sick leave	21 (9)	3 (3)
Full or part time	38 (17)	12 (13)
**Activity level of job**		
Hard physical work	62 (28)	33 (36)
Work with activity	97 (43)	39 (42)
Sedentary job	60 (27)	17 (19)
Not applicable	5 (2)	3 (3)

No difference in gender was found between patients who did (n = 318) and did not have (n = 35) complete data for the CPs calculations. However, patients with complete data were significantly younger (68.9 years (SD = 9.3)) than those who did not provide complete data for the CPs analysis (74.8 years (SD = 7.7); p < .001).

### Cutpoint calculations

The analysis resulted in different optimal CPs for patients with OA of the hip or knee. For average pain among patients with OA of the hip, the optimal CPs were (4,6), (1 to 4 is mild pain, > 4 to 6 is moderate pain, and > 6 to 10 is severe pain) because they had the lowest median rank of the ranked between-category-F-ratios, using Pillai's trace, Wilks' lambda, and Hotelling's trace statistics (Table 2). With this CPs classification, 22% of the sample (n = 50) had mild pain, 43% (n = 95) had moderate pain, and 35% (n = 79) had severe pain.

**Table 2:** Ranks for cut-point sets from the multivariate analysis of variance to determine optimal cutpoints using average pain intensity scores and the interference items from the Brief Pain Inventory among patients with OA of the hip (n = 224)

**Cutpoints**	**Pillai's trace**		**Wilk's lambda**		**Hotelling's trace**		
				
	Rank	F	Rank	F	Rank	F	Median rank
				
CPA3,5	5	8.33	5	9.26	5	10.21	5
CPA3,6	2	8.70	2	9.82	2	10.97	2
CPA3,7	6	7.96	6	9.04	6	10.15	6
CPA4,5	7	7.91	8	8.84	8	9.77	8
**CPA4,6**	**3**	**8.61**	**1**	**9.93**	**1**	**11.29**	**1**
CPA4,7	1	8.72	3	9.82	3	10.94	3
CPA5,6	8	7.85	7	8.91	7	9.98	7
CPA5,7	4	8.58	4	9.70	4	10.84	4

For patients with OA of the knee the optimal CPs for average pain were (4,7), (1 to 4 is mild pain, > 4 to 7 is moderate pain, and > 7 to 10 is severe pain) (Table 3). With this CP-based classification, 17% of the sample (n = 16) had mild pain, 65% (n = 61) had moderate pain, and 18% (n = 17) had severe pain. In further analysis of validity, the average pain CPs of (4,6) was chosen as the optimal CPs for patients with OA of the hip, while we chose CPs of (4,7) for patients with OA of the knee, in accordance with the above findings (Table 2 and 3).

**Table 3:** Ranks for cut-point sets from the multivariate analysis of variance to determine optimal cutpoints using average pain intensity scores and the interference items from the Brief Pain Inventory among patients with OA of the knee (n = 94)

**Cutpoints**	**Pillai's trace**		**Wilk's lambda**		**Hotelling's trace**		
				
	Rank	F	Rank	F	Rank	F	Median rank
				
CPA3,5	6	2.99	6	3.09	7	3.20	7
CPA3,6	7	2.91	7	3.08	5	3.25	5
CPA3,7	5	3.01	5	3.12	6	3.24	6
CPA4,5	4	3.28	3	3.69	3	4.09	3
CPA4,6	2	3.85	2	4.31	2	4.77	2
**CPA4,7**	**1**	**4.41**	**1**	**4.73**	**1**	**5.05**	**1**
CPA5,6	8	2.71	8	2.83	8	2.95	8
CPA5,7	3	3.34	4	3.37	4	3.40	4

### Demographic characteristics according to pain severity with the optimal cutpoints

When the three pain severity groups were compared, no differences were found in marital status, cohabitation, education level, employment status or activity level of job either among patients with OA of the hip or the knee (data not shown). However, patients with OA of the hip in the mild pain group were older than patients in the moderate pain group (p = 0.006). In addition, more women were in the severe pain group compared to the mild pain group (p = 0.003). There were no significant demographic differences according to pain severity groups among patients with OA of the knee.

### Pain characteristic and intensity according to pain severity with the optimal cutpoints

Patients with OA of the hip were in pain for almost 6.5 years, and had experienced mobility problems for almost 4 years before entering the waiting list. In contrast, patients with OA of the knee had experienced pain for 11 years and mobility problems for almost 7 years. No differences were found in the duration of pain or mobility problems, among the three pain severity groups (Table 4). Significant differences were found among the mild and severe groups on patients' ratings of the urgency of their surgery, both among those with OA of the hip and those with OA of the knee.

**Table 4:** Pain characteristics intensity scores according to the three pain severity groups, hip (n = 224) knee (n = 94), mean (SD)

	**Patients with OA of the hip**				**Patients with OA of the knee**			
		
**Pain characteristic**	**Mild pain 1 to 4** n = 50	**Moderate pain > 4 to 6** n = 95	**Severe pain 6 > to 10** n = 79	**P**	**Mild pain 1 to 4** n = 16	**Moderate pain > 4 to 7** n = 61	**Severe pain 7 > to 10** n = 17	**P**
		
Pain duration (years)	5.0 (5.1)	7.3 (7.7)	6.2 (6.3)	.15	14.4(14.3)	11.0 (10.5)	8.6 (8.6)	.32
Mobility problem (years)	3.2 (2.8)	3.5 (4.0)	4.6 (4.8)	.18	4.8 (4.8)	7.1 (7.9)	8.5 (10.7)	.47
Urgency for surgery (0–100)	60.6 (20.7)	67.4 (17.4)	80.2(13.9)	**< .001**^a^	55.4(15.4)	70.7 (18.5)	78.3(19.3)	**.002**^c^
**Pain Intensity Items from the Brief Pain Inventory**								
		
Pain now	2.3 (2.0)	4.8 (2.0)	6.8 (1.9)	**< .001**^b^	2.4 (1.6)	5.5 (1.8)	7.3 (2.3)	**< .001**^b^
Least Pain	1.5 (1.4)	3.1 (1.7)	4.9 (2.4)	**< .001**^b^	1.8 (1.5)	3.6 (1.6)	6.1 (2.6)	**< .001**^b^

Statistically significant p values are emphasized with bold face.

^a ^mild < severe, moderate < severe

^b ^mild < moderate < severe

^c ^mild < moderate, mild < severe

Significant differences in pain intensity scores were found among the three groups for both hip and knee on ratings of pain now and least pain (Table 4). For the pain intensity scores, post hoc contrasts showed significant differences among the three groups (mild < moderate < severe).

### Average pain with different cutpoints and pain on the SF-36 and WOMAC

Spearman's rank correlation of the BPI average pain intensity score recoded according to the eight possible cutpoint sets with the pain subscale on the WOMAC showed the highest correlation between the optimal CP for both hip (4,6) and knee (4,7) (Table 5). For hip patients, the correlation of categorized BPI average pain score with the bodily pain dimension on the SF-36 was highest with the optimal CPs, however tied with two other CPs (Table 5). For knee patients, the optimal CPs resulted in the second highest correlation with the bodily pain dimension on the SF-36 (Table 5).

**Table 5:** Spearman's rank correlation between the Brief Pain Inventory average pain intensity score with three severity levels according to variations in cutpoints (CPA) and corresponding scales of the SF-36 and WOMAC questionnaires for patients with OA of the hip or knee

	**Patients with OA of the hip**		**Patients with OA of the knee**	
		
	**WOMAC pain** n = 221	**SF-36 Bodily pain** n = 224	**WOMAC pain** n = 93	**SF-36 Bodily pain** n = 94
		
CPA 3,5	0.53	-0.51	0.49	-0.42
CPA 3,6	0.60	-0.54	0.50	-0.43
CPA 3,7	0.53	-0.48	0.50	-0.38
CPA 4,5	0.53	-0.50	0.51	-0.44
CPA 4,6	**0.61**	**-0.54**	0.54	-0.47
CPA 4,7	0.56	-0.49	**0.57**	**-0.46**
CPA 5,6	0.57	-0.54	0.51	-0.45
CPA 5,7	0.56	-0.53	0.54	-0.44

For all correlations, p < 0.001

Optimal cut-offs are bold-faced

## Discussion

This study is the first to determine CPs for mild, moderate, and severe pain in a sample of patients with OA of the hip or knee who are scheduled for joint replacement surgery. The optimal CPs for average pain, as assessed with the BPI and categorized as mild, moderate, and severe were CP(4,6) for patients with OA of the hip and (4,7) for those with OA of the knee, respectively. Hence, the optimal CPs differed between the two patient categories.

Another notable finding was that pain severity ratings were not associated with the duration of pain in either patient group. The optimal CPs for average pain resulted in the highest association with WOMAC pain for both patient categories and among the highest with the bodily pain scale of the SF-36, which supports the validity of the derived optimal CPs.

Previous work in non-cancer pain has reported different CPs for phantom limb pain (4,7), back pain (4,6), and for pain in general (3,6) [7], and that the optimal CPs for low back pain and OA are (5,8), and (5,7), respectively with the lower CPs of 5 (≤ 5 and > 5) being the most replicable and discriminative [8]. Hence the cutoffs seem to differ according to disease entities. Several reasons may account for the differences in CPs observed across these studies. For example the etiologies of the pain were different across the various studies, the demographic characteristics of the populations differed, and there were variations in whether ratings of average or worst pain were used to derive the CPs. In the present study, we used average pain intensity without a time dimension to derive CPs, as in previous studies [7,8]. Most recently, Paul and Zelman et al. using a homogenous sample of oncology outpatients with pain from bone metastasis, found that when a full range of CPs was tested, using both average and worst pain scores, the optimal CPs were (4,7) [5]. In addition, only three of the studies tested the full range of possible CPs [5,8,9]. The finding of different CPs for patients with OA of the hip and those with OA of the knee is new and may have several explanations. For example, it may be related to differences in the 'natural history' of the progression of OA of the hip or knee, differences in stage of the disease, differences in etiologies of the pain, or differences in demographic characteristics such as age or sex.

In the present study, we validated the BPI with the derived optimal CPs by assessing associations with the pain scales of the WOMAC and SF-36 questionnaires, using alternative CPs. The association of average pain with WOMAC pain was highest when using the derived optimal CPs, and among the highest with SF-36 bodily pain. In the framing of the items of the questionnaires, the time perspective for the BPI was the past 24 hours, for the WOMAC 48 hours, and for the SF-36 4 weeks. Therefore, a closer association of BPI average pain with WOMAC scores would be expected, and we would put more emphasis on this for the purpose of validation. This difference in time frame between the BPI and the WOMAC may possibly influence the results, however, we think the difference between 24 h and 48 h should be minor. Further, we asked the subjects to respond to questionnaires in relationship to the hip or knee joint that was to be replaced, however if subjects have multiple painful areas, they may have problems assessing the impact of one painful area on function.

In a previous study of patients with unspecified OA, who were undergoing treatment with cyclooxygenase-2 specific inhibitors and non-steroidal anti-inflammatory drugs [33], average pain exhibited good convergent validity, as assessed by the association with the pain subscale on the WOMAC. Hence, BPI appeared to be a useful measure of pain and pain interference and can be used to assess osteoarthritis pain and functioning.

In non-cancer pain, only the studies by Zelman et al. attempted to validate the severity categories using other outcome measures [5,8,9]. They reported that CPs calculations may be measure-dependent, for example they may be influenced by the differential sensitivity of the functional disability measure for levels of pain severity, or the correlation between pain severity and the reference measure. Further, the choice of pain item will influence the CPs, and it is claimed that pain-related interference may be most appropriate item for determining CPs for pain severity [12].

Worsening pain is the main indication for joint replacement surgery [34], although there are large variations in physicians' judgment regarding the priority of patients for joint replacement surgery. However, there is controversy in the literature about the optimal timing for joint replacement surgery in relationship to the duration of pain and mobility problems [26,27]. Therefore, we think the finding of little association between the pain severity ratings and the duration of pain and mobility problems in both the hip and knee patient groups in the present study is notable. Moreover, it is interesting to note that patients with OA of the knee reported longer duration of pain than patients with OA of the hip, which suggests a difference in the time-course of the conditions, or just a difference in selection of patients for surgery.

As noted in some previous reports, pain has been suggested to be a better predictor of disability and the need for surgery than does radiographic evidence of disease [1,35]. However, whether a structured pain assessment tool, such as the BPI, is useful in prioritizing patients for joint replacement surgery is not yet clear and needs to be assessed in longitudinal studies.

The patients in the present study were representative of the population of patients with OA who were scheduled for hip or knee joint replacement surgery, and they had similar WOMAC scores as patients in previous studies [2,26,27].

Several limitations of this study must be noted. Since patients were recruited by mail, the reasons why patients chose not to participate are not known. However, the patients who chose to participate were approximately 3.5 years younger then those who did not participate which may have biased the sample towards the reporting of higher pain intensity scores [36]. Another limitation was the lack of radiographic categorization of the extent of OA in these patients and the lack of information on comorbidity. In the present study, we did not consider the full range of possible CPs, but concentrated on a range from 3,5 to 5,7, in line with findings in previous reports [5,7-9]. Finally, patients were recruited from the public health care system, which does not necessarily allow for generalization to patients in the private health care system. However, in Norway the public health care system accounts for > 95% of hip and knee replacement surgery, hence we do not think this limits generalization of the results.

Some critique of the concept and use of CPs for pain should be raised. We have shown that the optimal CPs as derived with the standard methods differ between patient categories, which is also shown in previous studies. Therefore, there seems to be no universal optimal CPs, which makes such a classification difficult to use in practice. Because the CPs are based on the optimal group boundaries, this may not represent the best CPs for an individual patient [12], hence the concept of CPs may be difficult to use in a clinical setting. The categories mild, moderate and severe pain may be easier to interpret than the 11-level 0–10 scale of the BPI. Therefore, the categorization of BPI pain scores as mild, moderate or severe pain according to the optimal CPs may be useful in communication with patients about the interpretation of such scores. However, the severity levels of BPI average pain represent a reduction of the 11-level classification to a 3-level scale, which involves a considerable lack of information and also limits the choice of statistical methods for data analysis. Therefore, for research purposes it is probably better to use BPI on the continuous 0–10 scale. It also seems illogical to include those with a score of 0 (no pain) in a group with mild pain, and in some populations this would lead to large floor effects. Finally, optimal CPs may differ according to age, gender, race, or culture, which could represent another problem with optimal CPs. However, the latter has to our knowledge not been investigated.

## Conclusion

In summary, in the present study we have established optimal CPs for mild, moderate and severe pain among patients with OA of the hip or knee who were scheduled for joint replacement surgery. The optimal CPs differed between the patients categories. The associations between pain severity using these CPs support the validity of the optimal CPs, however, we think such a classification should be used cautiously. Whether the pain severity has a potential for clinical use or use in prioritization of patients for surgery could not be assessed in the present study, but may be a topic for future longitudinal studies.

## Competing interests

The author(s) declares that they have no competing interests.

## Authors' contributions

All authors participated in planning and design of the study. HK organised and supervised the data collection. HK and KS did the data analysis, drafted and revised the manuscript. BRH, NL and TR reviewed and commented on the manuscript. All authors read and approved the final manuscript

## Pre-publication history

The pre-publication history for this paper can be accessed here:


